# Beyond the Gut: A Case Report of Antibiotic-Induced Dysbiosis as a Hidden Cause of Chronic Insomnia

**DOI:** 10.7759/cureus.102143

**Published:** 2026-01-23

**Authors:** Ritesh Patel, Melissa Mangold, Ashok R Mandala

**Affiliations:** 1 Department of Medicine, Cooper University Hospital, Cooper Medical School of Rowan University, Camden, USA

**Keywords:** antibiotics, dysbiosis, gut-brain axis, gut microbiome, insomnia, probiotics

## Abstract

Insomnia is a common sleep disorder that affects many adults on a long-term or intermittent basis. While conventional management includes pharmacotherapy and cognitive behavioral therapy, emerging evidence highlights the gut microbiome as a critical regulator of sleep via the microbiome-gut-brain axis. Prolonged antibiotic exposure can lead to an alteration or imbalance in the structure, composition, and function of the gut microbial community, known as gut dysbiosis. Gut dysbiosis may disrupt neurotransmitter synthesis and circadian rhythm, contributing to refractory insomnia.

We report a 40-year-old man with early T-cell precursor acute lymphoblastic leukemia who underwent induction chemotherapy and allogeneic stem cell transplantation. Over two years, he received multiple prolonged antibiotic courses for infectious complications, resulting in significant cumulative exposure. Several months later, he developed severe insomnia, unresponsive to melatonin, mirtazapine, CBT-I, and multiple hypnotics, including zolpidem and lemborexant. Actigraphy revealed sleep latency of 90 minutes, total sleep time <4 hours, and sleep efficiency of 50-55%. Integrative evaluation demonstrated marked gut dysbiosis (low Firmicutes/Bacteroidetes ratio, overgrowth of Streptococcus, Klebsiella, Fusobacterium) and multiple micronutrient deficiencies. Targeted interventions, multistrain probiotics, fermented foods, high-fiber diet, and amino acid/vitamin supplementation, led to progressive improvement. At six months, the patient achieved restorative sleep without pharmacologic support, alongside improved mood, appetite, and functional status.

This case underscores the potential role of antibiotic-induced dysbiosis in chronic insomnia through disruption of microbial metabolites (short-chain fatty acids) and neurotransmitter pathways. Restoration of gut health may modulate circadian rhythm and sleep architecture, offering a novel adjunctive strategy for refractory insomnia.

Clinicians should consider gut microbiome assessment in persistent insomnia, particularly in patients with extensive antibiotic exposure. Further research is warranted to elucidate mechanisms and validate microbiome-targeted therapies.

## Introduction

Primary insomnia is a very common sleep disorder that affects approximately 10-15% adults chronically and up to one-third of individuals intermittently [1]. Beyond poor sleep, it impacts physical health, cognitive performance, mood, and overall quality of life [1]. Traditional management strategies for insomnia include pharmacotherapy, sleep hygiene, and cognitive behavioral therapy but clinicians often overlook systemic contributors to sleep regulation.

Growing evidence suggests that gut microbiomes are key modulators of neurophysiology through the gut-brain axis. Microbes produce substances such as short-chain fatty acids (SCFAs) and neurotransmitters like serotonin and gamma-aminobutyric acid (GABA), which regulate circadian rhythm and sleep architecture [2]. Disruption of microbial diversity is often described as dysbiosis. Gut dysbiosis can be triggered by a variety of factors, including imbalanced diet, antibiotic use, infections, chronic diseases, and other medications such as proton pump inhibitors, that disrupt the composition and function of the gut microbial community [3]. Gut dysbiosis has been associated with inflammation, metabolic dysfunction, and neurocognitive impairment [4].

Antibiotics are very essential in controlling infection, but they can profoundly alter the gut microbial composition by depleting valuable species including lactobacillus and bifidobacterium. These changes impair intestinal barrier integrity, enhance systemic inflammation, and possibly disrupt neurotransmitter synthesis which can lead to sleep disturbances [4]. There is growing research on the gut-brain axis, but the potential role of antibiotic-induced dysbiosis in chronic insomnia is not well established.

We report a case of severe treatment-resistant insomnia after recurrent antibiotics exposure, leading to gut dysbiosis and micronutrient deficiencies. The patient's clinical improvement after microbiome-targeted interventions highlights the importance of gut health evaluation in the management of chronic insomnia.

## Case presentation

A 40-year-old man with no prior comorbidities was diagnosed with early T-cell precursor acute lymphoblastic leukemia. He underwent induction chemotherapy (cyclophosphamide, adriamycin, dexamethasone, vincristine, methotrexate, cytarabine) for three cycles. During the induction period, the patient was taking levofloxacin for approximately three months. After remission, he underwent allogenic stem cell transplant following the conditioning regimen that included busulfan, fludarabine, and thiotepa. Post-transplant complications included ESBL Klebsiella pneumoniae bacteremia, treated with intravenous meropenem for one month. The patient remained in remission, which was confirmed by surveillance bone marrow biopsy every six months. He did not receive any chemotherapy after the transplant. Over the next two years, he experienced recurrent bacterial sinusitis mostly after acute viral infections. He required at least 15 courses of antibiotics (amoxicillin/clavulanic acid, doxycycline alternating courses), each course lasting approximately 10 days, resulting in significant cumulative antibiotic exposure. 

Three years after his transplant, the patient developed severe insomnia characterized by difficulty initiating and maintaining sleep. Initial evaluation revealed a PHQ-9 score of 3 (minimal depressive symptoms), a GAD-7 score of 3 (minimal anxiety), and an Insomnia Severity Index (ISI) score of 26 out of 28, consistent with severe insomnia. His lab work, which included complete blood count, comprehensive metabolic panel, vitamin B12, iron studies, thyroid profile, and vitamin D, was within normal limits. He was diagnosed with primary insomnia. 

Months 0-2

The patient was started on mirtazapine, which proved ineffective. Over-the-counter melatonin and cognitive behavioral therapy for insomnia (CBT-I) were also attempted during this period, without improvement. Actigraphy was performed. Actigraphy refers to a non-invasive method of monitoring and assessing rest-activity cycles, primarily used to estimate sleep patterns and circadian rhythms. Actigraphy showed prolonged sleep latency (~90 minutes), total sleep time under four hours, and sleep efficiency of 50-55%.

Month 3

Zolpidem, including the extended-release formulation, was initiated and provided short-term benefit. However, tolerance and withdrawal symptoms developed, leading to discontinuation after one month.

Months 4-5

Lemborexant (a dual orexin receptor antagonist) was prescribed, increasing sleep duration to 4-5 hours but causing significant morning drowsiness and constipation. However, the patient continued taking lemborexant for several months while simultaneously pursuing various alternative therapies.

Months 6-7

Despite several months of pharmacotherapy, insomnia persisted. The patient underwent one month of acupuncture therapy, which yielded no benefit.

Months 8-9

He then tried herbal remedies, including Ashwagandha and valerian, for two months, along with ayurvedic herbal oil massage, which provided only minimal symptom relief.

**Figure 1:**
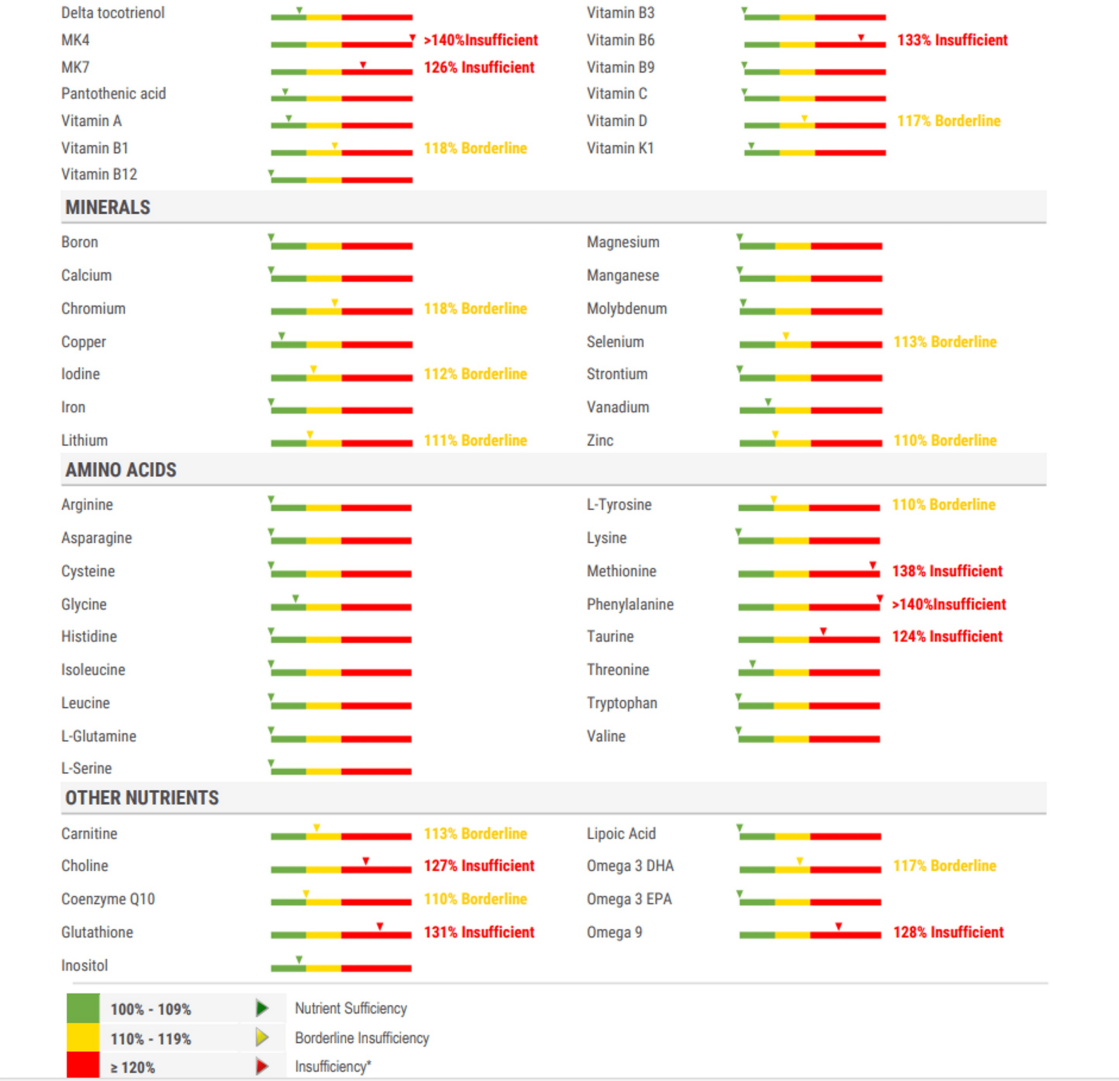
Cellular Micronutrient Assay

With his refractory insomnia, an integrative medicine evaluation was conducted. Integrative medicine is a patient-centered, evidence-informed approach that combines conventional biomedical treatments with complementary therapies. An integrative medicine specialist ordered a cellular micronutrient assay (CMA) (Figure 1) and GI microbial assay (Table 1). CMA showed multiple nutrient deficiencies, including vitamin K2 isoforms, riboflavin, pyridoxine, phenylalanine, methionine, taurine, choline, glutathione, and omega-9, with borderline deficiencies in thiamine, vitamin D, tyrosine, chromium, iodine, lithium, selenium, zinc, omega-3 DHA, carnitine, and coenzyme Q10. GI Microbial Map demonstrated significant dysbiosis without pathogenic organisms: elevated Bacteroides fragilis, Enterococcus, and Enterobacter; reduced Faecalibacterium prausnitzii; and overgrowth of Streptococcus, Klebsiella, Fusobacterium, and Prevotella. Both Firmicutes and Bacteroidetes were markedly increased, with a low Firmicutes/Bacteroidetes ratio (0.20). Intestinal health markers showed preserved digestion (Elastase-1 >750 μg/g), normal inflammation (calprotectin 16 μg/g), and adequate mucosal immunity (secretory IgA 1400 μg/g).

**Table 1:** GI Microbial Assay

Microbial Group	Finding	Clinical Significance
Bacterioides fragilis	Elevated	Commensal imbalance
Enterococcus spp.	Elevated	Commensal imbalance
Faecalibacterium prausnitzii	Reduced	Anti-inflammatory species
Firmicutes/Bacteroidetes ratio	Low (0.20)	Microbiome diversity shift
Streptococcus spp.	Overgrowth	Dysbiosis marker
Klebsiella spp.	Overgrowth	Inflammatory/autoimmune risk
Fusobacterium spp.	Overgrowth	Inflammatory/autoimmune risk
Prevotella spp.	Overgrowth	Inflammatory/autoimmune risk
Pathogenic bacteria/viruses	Absent	No acute infection
Opportunistic fungi/yeast	Absent	No fungal overgrowth

The patient was diagnosed with severe gut dysbiosis and functional micronutrient deficiencies, likely secondary to prolonged antibiotic exposure. This was thought to cause his insomnia via gut-brain axis disruption and impaired neurotransmitter synthesis. The patient started a multistrain probiotic formulation, fermented foods (kefir, kombucha, sauerkraut), a plant-based high-fiber diet with high polyphenols, omega-3/omega 9 fatty acid-rich foods, and targeted supplementation with deficient amino acids and vitamins. In two months, the patient noticed significant improvement in his sleep, where he did not require prescription hypnotics, but only melatonin and magnesium. At six months, he achieved restorative sleep without any supplements. Actigraphy at six months demonstrated sleep onset of approximately 30 minutes, total sleep time averaging 6-7 hours, and sleep efficiency of 75-85%, while maintaining an overall circadian rhythm. His ISI score was 4, consistent with no clinically significant insomnia. 

## Discussion

This case describes a possible association between antibiotic-induced gut dysbiosis and the onset of chronic insomnia, aligning with a growing understanding of the gut-brain axis in sleep regulation. The patient’s history of robust sleep prior to broad-spectrum antibiotic exposure, followed by the development of severe persistent insomnia, suggests a temporal and possibly causal relationship.

Role of gut microbiome in sleep

The gut microbiome has a significant influence on sleep regulation via the microbiome-gut-brain axis, which encompasses neural, endocrine, and immune pathways [5]. Microbial metabolites such as SCFAs, including butyrate, acetate, and propionate, play a central role in this process. SCFAs stimulate serotonin release from enterochromaffin cells, enhance melatonin receptor expression, and synchronize clock gene expression (Per, Cry, Bmal1), thereby influencing circadian rhythm and sleep homeostasis [5-7].

Gut microbes also produce neurotransmitters or their precursors, including GABA, which promotes sleep, and serotonin, dopamine, norepinephrine, orexin, and histamine, which promote wakefulness. These compounds act primarily through vagal signaling, even if they do not cross the blood-brain barrier [8,9]. Dysbiosis, characterized by reduced SCFA production and increased lipopolysaccharides, activates toll-like receptors and promotes systemic inflammation, leading to elevated pro-inflammatory cytokines and cortisol. This cascade disrupts NREM and REM sleep architecture and contributes to insomnia [9,10].

Animal studies provide strong evidence for these mechanisms. SCFA administration increased NREM sleep by up to 70% in rodents, while prebiotic supplementation improved NREM/REM sleep continuity and stress resilience [11,12]. These findings highlight the role of gut-derived metabolites and microbial signaling in sleep physiology.

Gut microbiome in diagnosis and treatment of sleep disorders

Understanding the gut microbiome’s role in sleep regulation has important diagnostic and therapeutic implications. Dysbiosis can lead to low levels of IL-6 and low SCFA levels, which may serve as biomarkers for refractory insomnia [13]. This may warrant microbiome profiling and functional assay in clinical evaluation of chronic insomnia.

Interventions targeting gut health, such as probiotics, prebiotics, and postbiotics, have shown potential to improve sleep latency, maintain deep NREM sleep, and reduce nocturnal awakenings in human trials [14]. Probiotic strains including Lactobacillus and Bifidobacterium have shown reductions in Pittsburgh Sleep Quality Index (PSQI) scores and improvements in EEG-based sleep parameters [15,16].

There are several limitations to this case observation. Although the ISI and actigraphy were utilized to provide objective assessment of sleep patterns, no additional objective outcome measures were included, which limits the depth of clinical evaluation and efficacy of the treatments. Furthermore, potential confounding factors, including comorbid conditions, were not fully controlled. Reliance on patient-reported improvements introduces subjectivity and possible recall bias. These constraints reduce generalizability and emphasize that the findings should be interpreted as hypothesis-generating rather than confirmatory.

## Conclusions

This case may suggest a possible association between antibiotic-induced gut dysbiosis and chronic insomnia, potentially mediated through disruption of the gut-brain axis. The patient’s improvement following microbiome-focused interventions, such as probiotics, fermented foods, and targeted nutrient supplementation, appears to indicate that restoring gut health could serve as an adjunctive approach for managing insomnia in select individuals. However, causality cannot be established from a single case, and these observations require further validation through controlled studies. Clinicians should consider gut health as one of several factors in refractory insomnia, while recognizing the need for additional research to clarify underlying mechanisms and evaluate the efficacy of microbiome-based therapies.
